# Supplementary material for: Population Dynamics and Neuronal Polyploidy in the Developing Neocortex
Source: Cereb Cortex Commun. 2020 Sep 7;1(1):tgaa063. doi: 10.1093/texcom/tgaa063 (PMC8152829; doi:10.1093/texcom/tgaa063)
Supplement: Suppl_Figures_tgaa063 [file suppl_figures_tgaa063.pdf]

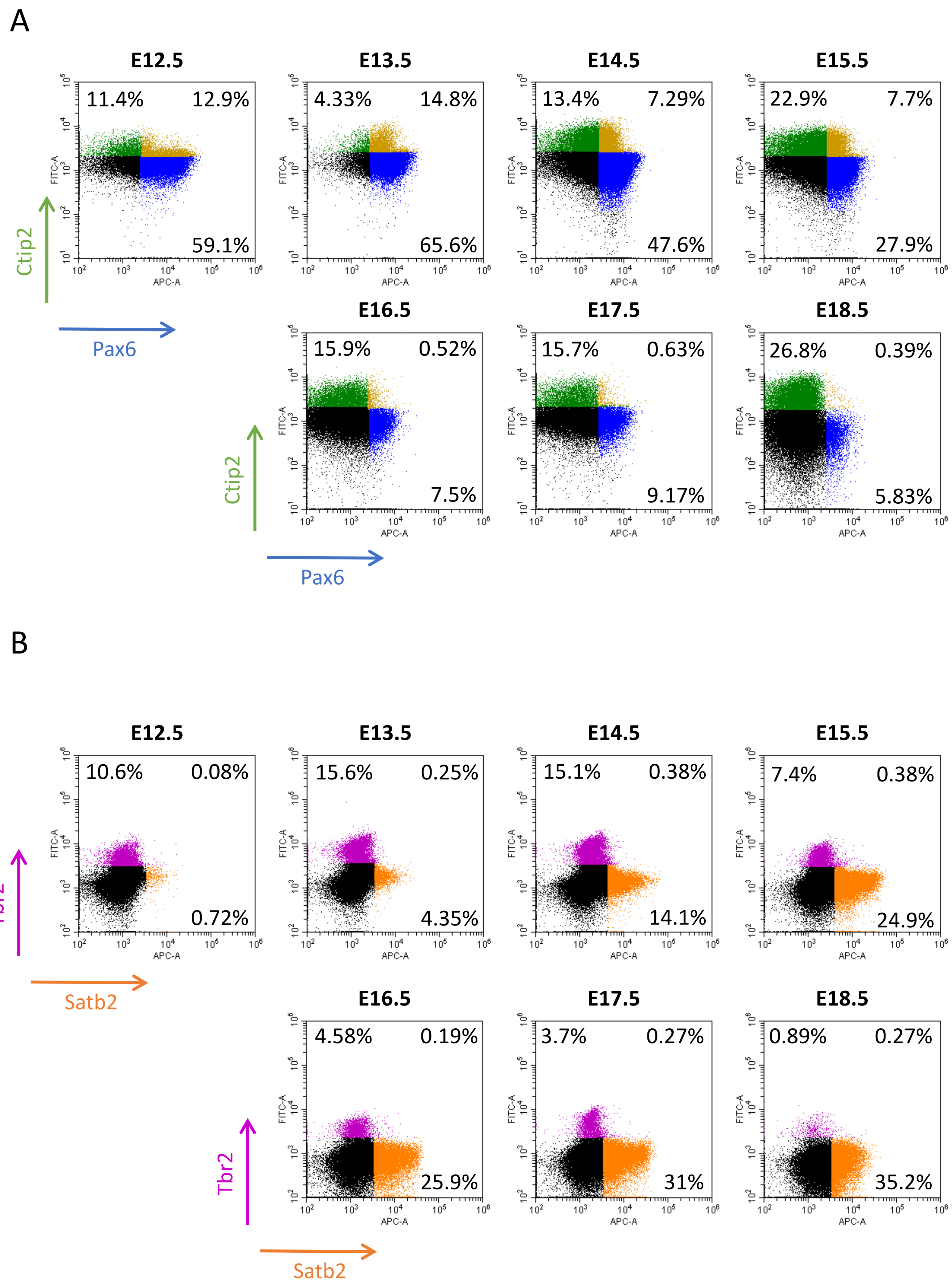

Sup Figure 1: Detailed analysis of Pax6/Ctip2 and Tbr2/Satb2 populations during neocortex development

Sup Figure 1: Detailed analysis of Pax6/Ctip2 and Tbr2/Satb2 double positive populations during neocortex development.

A: Representative dot plots for each developmental stage of Ctip2-Alexa fluor488 associated fluorescence intensity (vertical axis) versus Pax6-Alexa fluor647 associated fluorescence intensity (horizontal axis). Blue dots represent Pax6+ cells. Green dots represent Ctip2+ cells. Yellow dots represent double positive for Ctip2 and Pax6 (blue, upper right quadrant). The fraction of single and double positive cells is indicated in each quadrant.

B: Representative dot plots for each developmental stage of Tbr2-Alexa fluor488 associated fluorescence intensity (vertical axis) versus Satb2-Alexa fluor647 associated fluorescence intensity (horizontal axis). Purple dots represent Tbr2+ cells. Orange dots represent Satb2+ cells. The fraction of single and double positive cells is indicated in each quadrant.

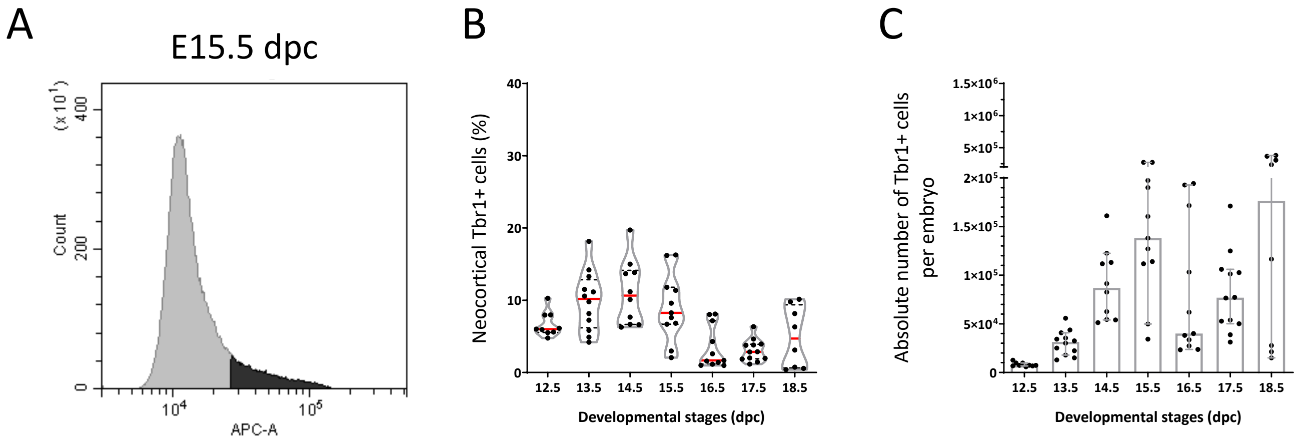

Sup Figure 2: Analysis of Tbr1+ cell population during neocortex development.

A: Histogram plot of cells from a E15.5 embryo showing Tbr1 associated Alexafluor 647 signal intensity detected on APC-A channel (Tbr1+ cells are in black).

B: Relative proportion of Tbr1+ cells at each indicated developmental stage. Each dot represents one embryo. Violin representation of the data displays the distribution (shape); the median (red line) and 1st and 3rd quartile (dotted black lines).

C: Absolute number of Tbr1+ cells in the neocortex per embryo at each developmental stage. The histogram bars correspond to the mean with 95% confidence interval error bars. Each dot represents the calculated value for one embryo.

A

sample id	age	PI volume	beads volume	lot beads concentration	acquired beads	acquired cells	Total cell number
P300-1	300 days	400 µl	50 µl	1 019 beads / µl	1 317	63 687	24 589 855
P300-2	300 days	400 µl	50 µl	1 019 beads / µl	1 498	63 019	21 391 964

B

stage	Pax6	Tbr2	Ctip2	Satb2	width (um)	Reference		
E10.5	65	10			100	Fei et al. Cell Reports		
E11	120	16			100	Alsio et al. PNAS		
E12.5	80	26			100	Lanctot et al. Cell Reports		
E12.5	120	39	23		100	Postel et al. BMC Bioinfo		
E13	190	56			100	Alsio et al. PNAS		
E13.5	110	120		21	100	Kischel et al. BMC Dev Biol		
E13.5	182	80			100	Fei et al. Cell Reports		
E13.5	155	106	13	43	100	Postel et al. BMC Bioinfo		
E14.5	150	120		65	100	Kischel et al. BMC Dev Biol		
E14.5	145	133	48	132	100	Postel et al. BMC Bioinfo		
E15	160	68			100	Alsio et al. PNAS		
E15	124	109	38	146	100	Postel et al. BMC Bioinfo		
E15.5	114	142	63	233	100	Postel et al. BMC Bioinfo		
E16	104	161	86	230	100	Postel et al. BMC Bioinfo		
E16.5				150	100	Kischel et al. BMC Dev Biol		
E16.5	69	130	105	251	100	Postel et al. BMC Bioinfo		
E16.5	80	150			100	Wang et al. Nature Neurosci.		
E16.5	175	110			100	Fei et al. Cell Reports		
E17	90				100	Alsio et al. PNAS		
E17.5			116	162	100	Alsio et al. PNAS		
E17.5	95	128	39	243	100	Postel et al. BMC Bioinfo		
E17.5	130	150			100	Yoon et al. Cell		
E18.5				180	100	Kischel et al. BMC Dev Biol		
E18.5	74	39	99	180	100	Postel et al. BMC Bioinfo		

C

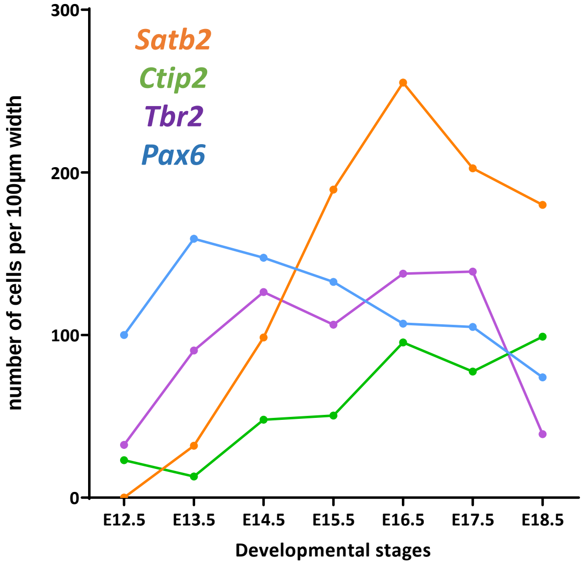

Sup figure 3: Comparison with published data at post-natal and pre-natal stages

Sup Figure 3: Comparison with published data at post-natal and pre-natal stages.

A: Raw data and calculation with the bead-based methods of the absolute number of cells for 2 distinct cortical samples from adult mice at 300 days post natal.

B: 2D data collected from a bibliographic survey.

C. Comparative dynamics of the 4 cell populations over the course of neocortex development: Pax6+ (blue), Tbr2+ (purple), Ctip2+ (green), Satb2+ (orange). Dots represent mean values from data in B, for each developmental stage.

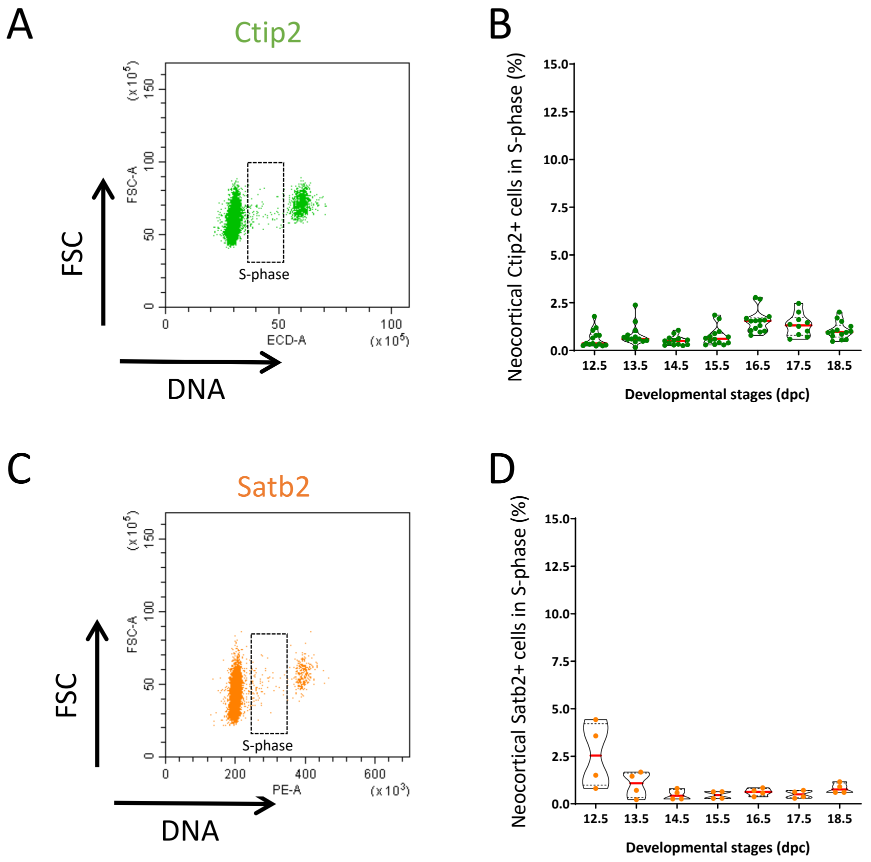

Sup Figure 4: Cell cycle parameters for the 2 subtypes of cortical neurons over the course of neocortex development.

A: Example of a dot plot of DNA content for Ctip2+ cells estimated by Propidium Iodide incorporation (horizontal axis) versus FSC relative cell size (vertical axis) for one embryo. Cells in S-phase are highlighted.

B: Distribution of the proportion of Ctip2+ cells in S-phase at each developmental stage. Each dot represents one embryo. Violin representation of the data displays the distribution (shape); the median (red line) and 1st and 3rd quartile (dotted black lines).

C: Example of a dot plot of DNA content for Satb2+ cells estimated by Propidium Iodide incorporation (horizontal axis) versus FSC relative cell size (vertical axis) for one embryo. Cells in S-phase are highlighted.

B: Distribution of the proportion of Satb2+ cells in S-phase at each developmental stage. Each dot represents one embryo. Violin representation of the data displays the distribution (shape); the median (red line) and 1st and 3rd quartile (dotted black lines).

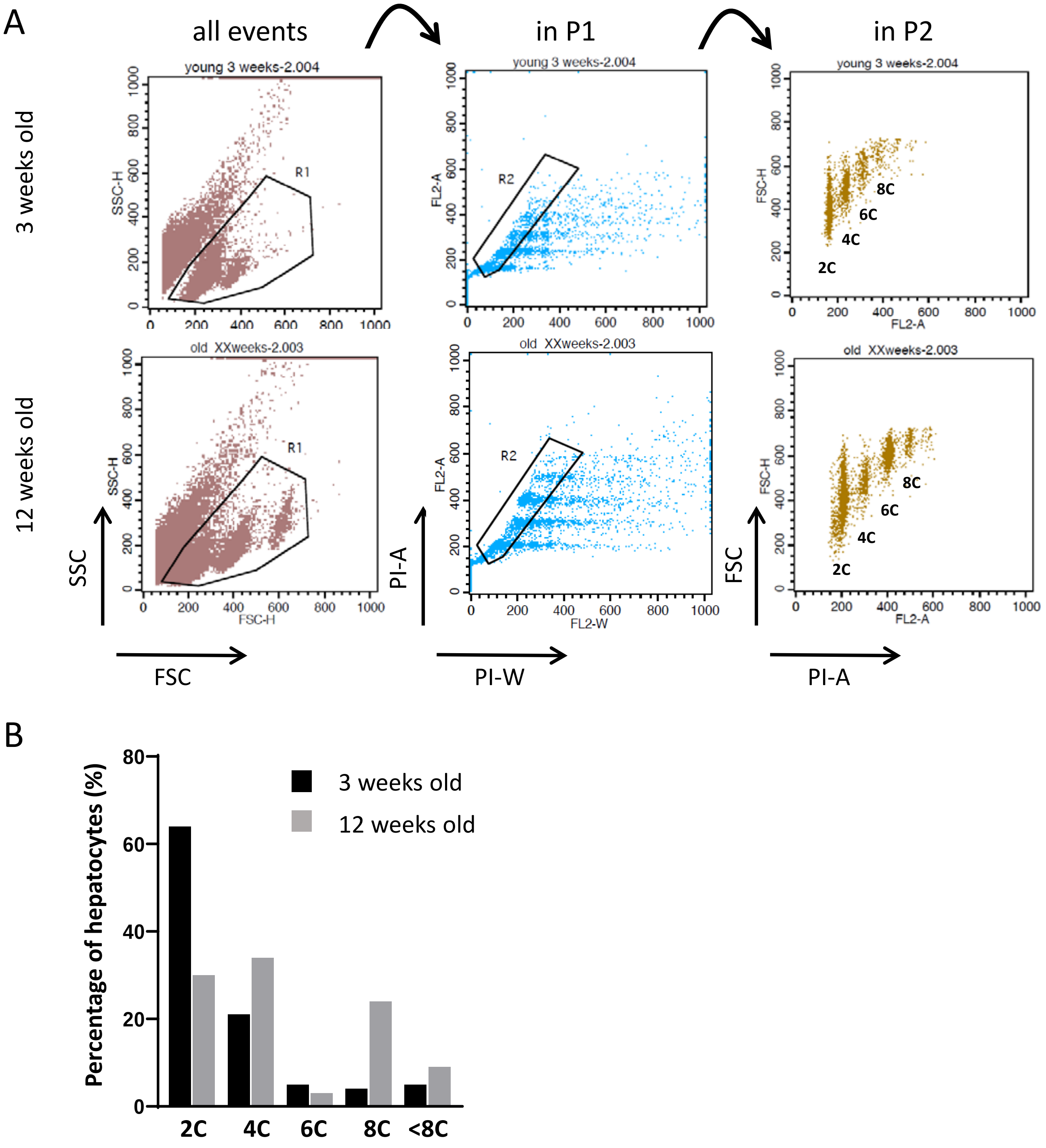

Sup Figure 5: Quantification of polyploid cells in adult liver using the flow cytometry procedure.

A: Side scatter (SSC) versus Forward Scatter (FSC) dot plot of all acquired events showing the gate R1 (left). Dot plot for Propidium Iodide Width signal intensity (PI-W) versus Propidium Iodide Area signal intensity (PI-A) of R1 gated events showing the gate R2 corresponding to the singlets retained for further analysis (middle). R1 and R2 gated cells plotted with FSC on vertical axis and PI fluorescence signal intensity (PI-H) on horizontal axis (right).

B: Fraction of hepatocytes (%) according to their DNA content. Bars represent one measurement of one sample.
